# Insulin-like Growth Factor 1 (IGF1) and Its Isoforms: Insights into the Mechanisms of Endometrial Cancer

**DOI:** 10.3390/cancers17010129

**Published:** 2025-01-03

**Authors:** Abdul Muzhill Hannaan Abdul Hafizz, Norfilza Mohd Mokthar, Reena Rahayu Md Zin, Nigel P. Mongan, Mohd Nazzary Mamat @ Yusof, Nirmala Chandralega Kampan, Kah Teik Chew, Mohamad Nasir Shafiee

**Affiliations:** 1 Department of Obstetrics and Gynaecology, Faculty of Medicine, Universiti Kebangsaan Malaysia, Cheras, Kuala Lumpur 56000, Malaysia; 2 Department of Physiology, Faculty of Medicine, Universiti Kebangsaan Malaysia, Cheras, Kuala Lumpur 56000, Malaysia; 3 Department of Pathology, Faculty of Medicine, Universiti Kebangsaan Malaysia, Cheras, Kuala Lumpur 56000, Malaysia; 4 Biodiscovery Institute, Faculty of Medicine and Health Sciences, The University of Nottingham, Sutton Bonington Campus, Loughborough LE12 5RD, UK

**Keywords:** endometrial cancer, insulin-like growth factor I, oestrogen signalling, tumour progression, cancer biomarkers

## Abstract

Endometrial cancer (EC) is a common gynaecological malignancy associated with metabolic dysfunctions such as obesity, diabetes and insulin resistance, as well as hormonal imbalances, particularly involving oestrogen and progesterone. These factors disrupt normal cellular metabolism, heightening the risk of developing endometrioid EC (EEC), the most prevalent subtype of EC. The insulin-like growth factor-1 (IGF1) pathway, a key regulator of growth, metabolism, and organ function, is implicated in EC progression. Recent research highlights the distinct roles of IGF1 isoforms, including IGF1-Ea, IGF1-Eb, and IGF1-Ec, in promoting tumour growth, metastasis, and hormone signalling interactions, particularly with oestrogen. This review examines the function and clinical significance of IGF-1 isoforms, emphasising their mechanisms in gynaecological physiology and their contributions to EC pathogenesis. Evidence from other cancers further underscores the relevance of IGF1 isoforms in driving tumour behaviours, offering valuable insights into their potential as biomarkers and therapeutic targets. Understanding these mechanisms provides opportunities for novel approaches to the prevention, diagnosis, and treatment of EC, improving patient outcomes and advancing the broader field of hormone-driven cancers.

## Simple Summary

Metabolic conditions such as obesity, diabetes, and insulin resistance, combined with hormonal imbalances, can disrupt the body’s natural growth regulation systems, including the insulin-like growth factor-1 (IGF1) pathway. These disruptions significantly increase the risk of endometrial cancer (EC), a cancer that develops in the lining of the uterus. This review delves into the roles of IGF1 and its variants; IGF1-Ea, IGF1-Eb, and IGF1-Ec, and their interactions with hormones like oestrogen in driving tumour growth and spread. By unravelling these connections, we aim to identify innovative strategies for preventing and treating this cancer, bringing hope for improved patient outcomes and advancing research in hormone-driven cancers.

## 1. Introduction

Endometrial cancer (EC) arises from the uterine lining and is influenced by multifaceted factors, including metabolic dysfunctions such as abdominal obesity, hyperglycaemia, hypertension, and atherogenic dyslipidaemia [1,2]. Visceral fat deposition disrupts insulinotropic and anti-inflammatory cytokines, leading to insulin resistance and heightened susceptibility to developing EC in postmenopausal women [1]. Hormonal imbalances significantly impact endometrial health, potentially increasing the risk of polycystic ovary syndrome (PCOS), endometriosis, and EC [2,3]. Molecular and hormonal variations contribute to EC aetiology, with unopposed oestrogen and endocrine system dysfunction being notable risk factors [4,5].

Recent studies have explored changes in circulating insulin, insulin-like growth factors (IGFs), insulin-like growth factor binding proteins (IGFBPs), inflammatory cytokines, adipokines, and sex hormones in EC [1,2,6,7]. IGF1 has been identified as a central hub gene, alongside others like CCNA2, CDK1, CCND1, FGF2, BCL2, and VEGFA, which are potential targets for gynaecological cancer prevention and early intervention [8]. These genes regulate critical intracellular signalling pathways, including the phosphatidylinositol 3-kinase (PI3K)/AKT and mitogen-activated protein kinase (MAPK) pathways, specifically the extracellular signal-regulated kinase (ERK) pathway, which trigger cancer development [7,8]. Building on these molecular insights, current therapeutic strategies for EC include surgery, chemotherapy, and radiotherapy [9]. Chemotherapy with agents like paclitaxel and carboplatin remains the standard approach, especially for advanced or recurrent cases [10,11]. Emerging therapies, such as IGF1R and mTOR inhibitors, target these oncogenic pathways, while hormonal therapies and immune checkpoint inhibitors like pembrolizumab are actively being studied [12,13,14,15,16]. These advancements emphasise the critical need to further investigate IGF1 isoforms, and this review explores their roles in EC pathogenesis and their potential as novel therapeutic targets.

Further research is warranted to explore epigenetic pathways involving microRNA and sex steroid profiling in EC [5]. Investigating these pathways is essential for understanding the interplay between metabolic and epigenetic factors in endocrine-related tumour development. These pathways may elucidate how behavioural and microenvironmental stimuli induce changes in cell function, thereby altering the functionality of IGF1 and its associated genes. Metabolic syndromes, hormonal imbalances, and sex steroid milieu have been linked to IGF1 signalling activation in endocrine-related tumour progression [6,17,18]. IGF1, ubiquitously expressed with complex metabolic regulatory roles, has emerged as a promising therapeutic target for cancer [18,19]. The objective of the review is to explore the association between IGF1 and its isoforms in the pathogenesis of EC, providing insights into potential novel targets for its treatment.

## 2. Insulin-like Growth Factor 1 (IGF1)

The mature IGF1 protein, also known as somatomedin C, consists of a 70-amino-acid single-chain basic polypeptide with a molecular weight of approximately 7.5 kDa. It exerts tissue-specific effects through the IGF1 receptor (IGF1R), playing a crucial role in controlling the IGF hormonal network. Human IGF1 is primarily produced in the liver and released into the bloodstream under the regulation of growth hormone (GH) [17,19]. Upon binding to the α subunit of IGF1R, the IGF1 ligand induces a conformational change, activating receptor tyrosine kinase activity [18]. Phosphorylation of IGF1R further triggers signal transmission across the Ras(Rat Sarcoma)–Raf(Rapidly Accelerated Fibrosarcoma)–MAPK and PI3K-AKT pathways, influencing processes such as cell proliferation, differentiation, apoptosis, and carcinogenesis [20,21].

**Figure 1:**
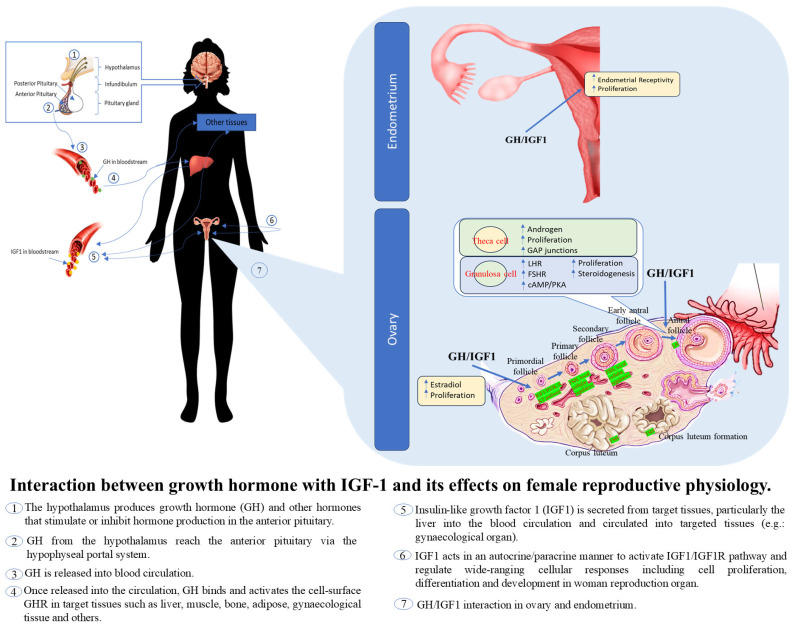
Effect of GH and IGF1 secretion in relation to women’s reproductive system physiology. The pulsatile GH secretion stimulates the production and secretion of IGF1 from the liver, which is the primary source of circulating IGF1 and the GH/IGF1 effect.

The GH-IGF1 signalling pathway plays a critical role in female reproductive physiology, impacting processes such as folliculogenesis, ovarian steroidogenesis, and embryo implantation [7]. Figure 1 illustrates how the pulsatile release of GH triggers the liver to produce and release IGF1, which then affects the female reproductive system. Cellular IGF1 expression extends beyond surface receptors, with emerging recognition of nuclear functions for both IGF1 and its receptor [19]. Investigating IGF1 genes and their isoforms across various contexts is essential to better understand their roles in diseases, including EC. IGF1R, a key player, regulates gene expression by modulating chromatin remodelling proteins and participating in DNA damage-tolerance mechanisms [19,22]. Further research is needed to explore the potential of specific antibodies for IGF1R internalisation and degradation.

As IGF1 alternative splicing (AS) has emerged as a potential therapeutic target in oncology, it is important to gain a deeper understanding of the associated signalling components. The IGF1 gene generates numerous isoforms, each potentially serving a distinct physiological function. This review elucidates the significance of IGF1 isoform expression in physiology and pathogenesis, in addition to the mechanisms by which IGF1 spliced variants contribute to tumour biology and their potential as a target in EC research.

## 3. The IGF1 and Cancer Development

As the central dogma of IGF1 applies to most human malignancies, the IGF1 hormonal network (including ligands, cell-surface receptors, and IGF-binding proteins) can react through different autocrine, paracrine, juxtracrine, or endocrine signalling pathways [18]. Recent findings have revealed that IGF1 is secreted locally by various cell types, depending on cancer cell behaviours rather than solely from the liver [18]. Additionally, alterations in its receptor (IGF1R) have attracted significant attention in malignant cells [19,22], with IGF1R overexpression and AKT pathway activation linked to early stages of endometrial carcinogenesis in hyperplastic endometrium [23]. Interest in understanding the biological control linked with IGF1 has been driven by both theoretical and practical elements of clinical oncology. Dysregulation of the IGF1 pathway is associated with malignancies, including less common types [7]. Epidemiological studies consistently link elevated circulating IGF1 levels to primary malignancies such as lung, breast, colorectal, ovarian, and EC [6,7,18,24,25]. Our comprehension of IGF1 regulation in gynaecological cancer biology is still lacking. Christopoulos et al. (2015) have brought attention to the diverse expression patterns of IGF1 isoforms across various types of cancer in recent studies [26]. The diverse expression patterns of IGF1 isoforms in different types of cancer are significant, as IGF1 is moderately linked with an increased risk of total cancer in both men and women, with divergent associations observed for specific cancer types, as highlighted by Qian and Huo (2020) [24]. These isoforms may impact the IGF1 signalling pathway, systemic circulation, and organ-specific interactions. To deepen our understanding, it is imperative to investigate the mechanisms governing local IGF1 synthesis and its isoforms within the cellular microenvironment. Understanding these complexities will contribute to advancing cancer research and clinical practice.

## 4. Therapeutic Potential and Challenges of IGF1 Targeting in Cancer

The IGF1 signalling pathway is a vital modulator of cancer progression, including EC, rendering it a compelling therapeutic target. Preclinical studies demonstrate that IGF1R inhibitors decrease tumour development, promote apoptosis, and interfere with critical pathways such as PI3K/AKT and MAPK/ERK, which are essential for tumour survival and proliferation [27,28,29]. Moreover, combination treatments that target IGF1R alongside agents like mTOR inhibitors have demonstrated enhanced efficiency in preclinical models, suggesting potential strategies to improve treatment outcomes [21,28]. However, clinical trials investigating IGF1R inhibitors, such as figitumumab and ganitumab, have produced mixed results, with modest responses and significant metabolic side-effects, including hyperglycaemia, due to IGF’s role in glucose metabolism [30,31,32].

Several challenges hinder the clinical success of IGF1-targetted therapies. The structural similarity between IGF1R and the insulin receptor (INSR) leads to off-target effects, exacerbating metabolic issues [18,32]. Furthermore, cancer cells often activate compensatory pathways, such as HER2 or RAS/MAPK signalling, in response to IGF1R inhibition, diminishing therapeutic efficiency [33]. The heterogeneity of EC, including variations in IGF1 expression across tumour subtypes, further complicates the development of effective therapies and underscores the need for predictive biomarkers to identify patients most likely to benefit from IGF1-targeted treatments [34,35].

The tumour microenvironment (TME), particularly hypoxia, significantly influences IGF1 signalling, posing challenges in cancer therapy [36,37]. Hypoxia-inducible factors (HIFs) activate pathways such as PI3K/AKT and MAPK, driving tumour progression and resistance to treatment [36,38]. IGF1 has been shown to respond to hypoxic conditions, contributing to extracellular matrix remodelling, tumour invasion, and metastasis [37]. Additionally, hypoxia enhances IGF1-mediated VEGF expression, promoting angiogenesis and sustaining tumour growth [39]. While the role of IGF1 isoforms in hypoxic conditions remains unexplored, future research could investigate their potential contributions to tumour progression and therapeutic resistance in the TME, offering new insights into cancer biology.

Future research should focus on developing highly selective IGF1R inhibitors to minimise off-target effects, as well as exploring combination therapies to counteract compensatory mechanisms. Incorporating biomarkers for patient stratification will enhance therapeutic precision, ensuring that treatments are tailored to the molecular characteristics of individual tumours. Furthermore, understanding the distinct roles of IGF1 isoforms in tumour biology may provide insight into optimising IGF1-targeted therapies overcoming resistance mechanisms.

## 5. Circulating IGF1 Levels and EC Development

The relationship between circulating IGF1 and EC risk remains contentious, with studies yielding contradictory results. Ayabe’s research team initially suggested that elevated IGF1 and reduced IGFBP1 levels are associated with an increased risk of postmenopausal EC, highlighting IGF1′s potential role in carcinogenesis [40]. Conversely, Petridou et al. (2003) linked EC to elevated IGF2 and decreased IGF1 levels, adding complexity to the narrative [41]. Gunter et al. (2008) further nuanced this understanding by identifying an inverse association between IGF1 and EEC, particularly in obese women [42]. Despite these findings, several studies have reported no significant differences in serum IGF1 concentrations between EC patients and controls [43,44,45]. McGrath et al. (2011) did not find any impact of IGF1 genetic variability on EC occurrence but noted an inverse association of IGF2 with EC risk, contradicting the earlier findings of Petridou et al. (2003) [41,46]. In addition, free IGF1 plasma levels were significantly lower in EC patients with myometrial invasion compared to those without myometrial invasion [47]. This array of conflicting results underscores the complexity of the IGF system in EC pathogenesis and indicates the need for further research to elucidate these relationships. While conflicting results exist regarding the role of IGF1 in EC development, this review delves into its potential mechanisms and contexts.

Impaired glucose tolerance and hyperinsulinaemia are also implicated in increasing EC risk among women [1,2,48]. Notably, women with type II diabetes exhibit significantly higher serum IGF1 levels compared to the general population [49]. Ethnic differences, hormonal factors, and the effects of hormone replacement therapy (HRT) further complicate this relationship. Some studies have related the elevated IGF1 serum with different ethnic in their studies [50,51]. Nevertheless, some of the cited above-mentioned studies failed to account for hormone replacement therapy (HRT) or hormonal contraceptive use. For instance, oral HRT has been shown to significantly reduce serum IGF1 levels, likely due to the direct inhibition of IGF1 production in the liver by orally administered oestrogen [17,52,53]. In addition, elevated serum IGF1 was also reported in PCOS, which significantly increased the EC risk [6]. Despite these insights, the precise mechanisms through which IGF1 contributes to endometrial carcinogenesis remain unclear, necessitating further research to elucidate these pathways.

## 6. Alternative Splicing (AS)—A Phenomenon in Physiology and Pathophysiology in EC

Alternative splicing (AS) has broad implications across disciplines. In medicine, understanding AS regulation sheds light on physiological and pathophysiological mechanisms, aiding diagnostic marker identification and therapeutic target discovery [54,55]. Aberrant AS events are linked to cancer development, but the impact of genetic variants on EC risk remains uncertain. AS generates distinct protein isoforms, modulating protein function in various physiological conditions [55]. Complex interactions between spliceosomal machinery and regulatory components, including splicing enhancers and silencers, contribute to proteome diversity [54,55]. They play a vital role in enhancing the functional diversity of the proteome by enabling cells to generate proteins with unique structural and functional characteristics.

Pre-mRNA splicing, a critical process orchestrated by the spliceosome, is fundamental in maintaining cellular function and plays a pivotal role in disease and cancer progression. Dysregulation of splicing factors, including Splicing Factor 3B Subunit 1 (SF3B1), contributes to abnormal mature transcripts associated with tumorigenesis [56]. This highlights the importance of understanding splicing mechanisms at a tissue-specific level, as researchers are now delving into these mechanisms to uncover novel biomarkers for disease [57]. Recent studies on SP3B1 inhibitors and an AS signature model for EC prognosis provide valuable insights [56]. Aberrant AS of IGF1 isoforms may alter cellular functions, disrupting keys pathways like PI3K/AKT and MAPK/ERK, driving cell proliferation, survival, and migration in EC. These splicing events highlight the critical role of IGF1 isoforms in shaping the tumour microenvironment, as discussed in detail in the next section.

## 7. IGF1 Gene and Its Isoforms

**Figure 2:**
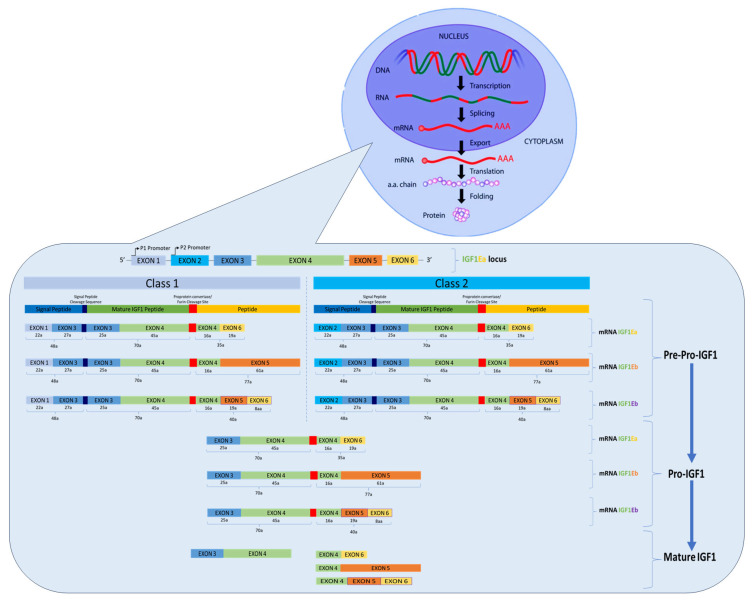
Schematic representation of the human IGF1 gene. The IGF1 gene consists of six exons (coloured boxes) separated by five introns. Exons 1 and 2 contain multiple transcription initiation sites. Translation initiation codons (AUG) are located at exons 1, 2, and 3. Exons 1, 2, and 3 encode the signal peptide of precursor IGF1 in the cytoplasm. Exons 5 and 6 generate three distinct isoforms: IGF1-Ea, IGF1-Eb, and IGF1-Ec.

The human IGF1 gene, located on chromosome 12 (12q22–12q24), comprises six exons and five long introns, with two transcriptional promoters (P1 and P2) [58]. As depicted in Figure 2, precursor IGF1 polypeptides include the signal peptide, mature IGF1, and the E peptide. Two classes of mRNA variation arise: class 1 transcripts initiate from exon 1 (P1), while class 2 transcripts use exon 2 (P2). Post-translational modifications, including proteolytic processing and glycosylation, yield diverse pre-pro-peptide transcript variants [58]. Exons 3 and 4 encode the conserved mature IGF1 peptide. Exon 3 contributes to the signal peptide, while exon 4 includes part of the mature peptide and the E-amino-terminal region shared by all IGF1 mRNAs. Alternative splicing of exons 5 and 6 generates three carboxyl(C)-terminal E peptides and distinct 3′UTR: Ea (exon 6, the most abundant transcript), Eb (exon 5), and Ec (fragments of exons 5 and 6, also known as mechano-growth factor (MCF)).

The human IGF1 gene undergoes alternative splicing, resulting in three protein isoforms (pro-IGF1-A, pro-IGF1-B, and pro-IGF1-C). Post-translational cleavage eliminates the signal and E peptide, yielding mature IGF1 and E-peptides. These isoforms, secreted from cells, act as obligate ligands for IGF1R phosphorylation, driving mitogenic signalling in targeted cells. Their distinct stabilities, binding partners, and activities suggest tissue-specific roles [58]. Understanding the functional effects of IGF1 gene alternative splicing may reveal new molecular mechanisms of carcinogenesis and guide anti-cancer therapeutic strategies.

## 8. IGF1 Isoforms in Physiology

IGF1, a network of cellular and secreted proteins, acts as a potent mitogen with proliferative and anti-apoptotic effects. While liver hepatocytes are the primary source of IGF1 secretion, other organs also contribute to its biosynthesis. Notably, healthy mature hepatocytes express low levels of IGF1R compared to other tissues [18]. Physiologically, IGF1 isoforms are predominantly found in the liver, skeletal muscles, and adipose tissue [17]. However, the precise roles of local versus circulating IGF1 fractions remain incompletely understood.

Growing evidence highlights distinct roles for IGF1 isoforms (IGF1-Ea, IGF1-Eb, and IGF1-Ec) in skeletal muscle development, regeneration, and maintenance [58]. Following muscle damage, IGF1-Eb mRNA is initially upregulated, promoting myoblast proliferation, while subsequent IGF1-Ea upregulation is associated with myofiber differentiation [58,59]. These locally acting isoforms also exhibit neuroprotective properties and shield cardiomyocytes from stress [60]. Notably, IGF1-Ea mRNA prevails in multiple organs, suggesting an organ-specific regulatory mechanism [58]. Furthermore, the E-peptides may influence the cellular entry of mature IGF1, adding another layer of complexity to their function [61]. The regulation of IGF1 isoforms in systemic protection mechanisms, including tissue repair, may provide insights into their role in EC development.

The relative abundance of IGF1 isoforms may stem from variations in their maturation. The pro-receptor form of IGF1 undergoes proteolytic cleavage in the Golgi compartment, controlled by the convertase furin. Human muscle and liver protein lysates confirm that pro-IGF-1Ec can be cleaved to yield the free Ec peptide [62]. Notably, IGF1-Ec also plays a significant role in bone development and repair [63]. Post-translational modifications of the IGF1-Ec pro-peptide likely occur and are influenced by the surrounding cell microenvironment.

The existence of different IGF1 isoforms raises questions about their distinct biological activities and tissue-specific control of IGF1 production. Recent research suggests that IGF1-Ea and IGF1-Eb hold promise as therapeutic agents for preventing muscular weakness [64]. These isoforms enhance the autophagy/lysosome system, counteracting age-related changes, and increase Peroxisome Proliferator-Activated Receptor Gamma Coactivator 1-Aplha (PGC1-α) expression, regulating mitochondrial function and inflammation associated with ageing. Furthermore, IGF1-Ea’s modulation of endothelial-like tubular structures may have clinical implications for regenerative therapies [65].

The IGF1 system, which exhibits expression variations influenced by menstrual cycle phases, was initially described in endometrial function in 1998 [66]. Both IGF1 signalling and ERα-mediated responses are essential for a complete uterine response to oestrogen and growth [67]. While Milingos et al. (2011) reported IGF1 isoform expression in endometrial tissues, the current literature lacks data on isoform variability in normal endometrial physiology [68]. Future research on IGF1 isoforms, including their role during menstrual cycles, holds promise for improving clinical outcomes in gynaecological fields.

## 9. IGF1 Isoforms in Pathophysiology and Cancer Biology

The involvement of IGF1 signalling pathways in endocrinology, paediatrics, ageing, and oncology has captured researchers’ attention. Alterations in IGF1 isoforms may persistently impact the cellular milieu, potentially leading to mutations in target genes that initiate cancer development. The growth of abnormal cells may indeed be related to specific IGF1 isoforms. In cardiac remodelling, the delivery of IGF1-Ea pro-peptide has been shown to improve outcomes after myocardial infarction by enhancing cardiomyocyte survival, promoting regeneration, and modulating immune cell recruitment and cytokine expression [60].

The IGF1 isoforms exhibit diverse roles in various physiological contexts. IGF1-Eb is crucial for mitochondrial protein synthesis in skeletal muscle, and its decreased mRNA levels may contribute to impaired protein synthesis in obese individuals [69]. Meanwhile, IGF1-Ec emerges as a potential therapeutic target in osteosarcoma due to its distinct physiological role compared to IGF1 [63]. Notably, IGF1-Ec is independently associated with reduced skeletal muscle mass, BMI, and gender [69]. In endometriosis, all IGF1 isoforms are expressed in eutopic endometrium and ovarian endometrioma, but their levels significantly decrease in endometriotic cysts [70]. This reduction correlates with disease status and fibrotic tissue presence in late-stage endometriosis. Additionally, IGF1-Ec (MGF) expression is preferentially observed in glandular epithelial cells of ectopic endometrium, suggesting its role in tissue remodelling, fibrosis, and cellular proliferation [68]. These functions may contribute to the maintenance and progression of ectopic endometrial lesions, highlighting IGF1-Ec as a potential therapeutic target or biomarker for late-stage endometriosis.

Research spanning nearly two decades has linked IGF1 to the onset of various malignancies. Investigations into IGF1 mRNAs and pro-peptide variants aim to unravel their biological significance in carcinogenesis. Most examined cancers exhibit a prevalence of class I transcript expression over class II, although some studies report minor differences in colorectal cancer (CRC) [71,72]. Indeed, EC may aberrantly express each component of IGF1 isoforms. As a growing number of studies have sought to decipher the molecular mechanisms underlying the association of IGF1 systems with EC, the AS of IGF1 has emerged as the current area of cancer research interest. IGF1-Ea predominates in CRC and cervical cancer (CC), while cultured cell lines also show the presence of IGF1-Ea in EC, breast cancer (BC), and melanoma [26,71,72,73,74]. The other mRNA isoforms, IGF1-Eb and IGF1-Ec, and their corresponding pro-peptides may be overexpressed in different human cancers [26,58,75,76].

IGF1-Ec is believed to play a mitogenic role in tumours, supported by research on human cancer cell lines [26,58,72,75,76,77,78]. In contrast, the role of IGF1-Eb remains controversial, with conflicting evidence suggesting both anti-cancer and cancer-promoting effects [75,79]. Some studies report increased affinity of certain IGF1 isoforms to IGF1R, implicating them in cancer development [63,79]. However, contradictory findings indicate that IGF1 variants can act independently of IGF1R and INSR [26,58].

In BC, the hormonal status (Estrogen Receptor (ER)-positive and ER-negative) affects how IGF1-Ea and IGF1-Ec influence cell proliferation, progression, and resistance to therapy through interaction with ER, particularly in ER-positive cases [75,76] The absence of IGF1-Eb transcripts may enhance cancer cell aggressiveness, while studies debate its role as an anti-cancer factor by inhibiting BC cell growth, invasion, and angiogenesis [75], while in CC, human papillomaviruses (HPVs) interact with IGF axis components, potentially influencing spliceosome elements [74]. IGF1-Eb exhibits pro-proliferative activity in CC, with complex nucleolar/nuclear localisation [79,80]. Additionally, hepatocellular carcinoma involvement depends on factors like inflammation and hepatotropic virus infection, necessitating further research [81]. IGF1 isoforms also impact lung cancer (LC), osteosarcoma (OS), prostate cancer (PC), thyroid cancer (TC) and other neuroendocrine neoplasms where IGF1-Ec plays an oncogenic role by stimulating proliferation, progression through epithelial to mesenchymal transition (EMT), and metastasis via ERK1/ERK2 activation [26,58,74,75,77,82]. All the related findings of IGF1 isoforms in various cancer research are summarised in Table 1.

## 10. Potential Role of IGF1 Isoforms in the Biology of EC

Notably, the IGF1 level is not the only factor that activates the IGF1/IGF1R pathway. Although research on the effects of abnormal post-translational modifications in IGF1 precursors in EC is limited, these modifications have been reported to contribute to the activity of IGF1 [26,58,70,86]; the findings are summarised in Table 2. Stavropoulos et al. (2020) had retrospectively reported that IGF-1Ec immunohistochemical staining was significantly higher in 99 EC archival specimens compared to normal endometrium and endometrium hyperplasia (EH) (*p* < 0.05) [86]. IGF1-Ec has been observed to antagonise PTEN’s tumour-suppressive functions by activating downstream pathways such as PI3K/AKT and MAPK, which promote cell proliferation and inhibit apoptosis [86,87]. Similarly, IGF1 signalling can attenuate p53-medated cell cycle arrest and apoptosis, enhancing tumour growth [88]. Survivin, an apoptosis inhibitor, is upregulated by IGF1 signalling, contributing to therapy resistance and sustained tumour survival [89].

Nevertheless, this in vivo study exclusively identified IGF1-Ec isoforms and did not compare them to other IGF1 variants, while several studies only concerned the KLE cell line (non-endometrioid EC subtype), which did not represent the major subtype of EC [26,68]. Intriguingly, Christopoulos et al. (2015) demonstrated that the IGF1-Ea and IGF1-Ec isoforms are expressed at the highest levels in KLE cell lines compared to other cancer cell lines studied [26]. The distinct potential molecular functions of IGF1 and E peptides, particularly those of its various classes in EC, should be investigated, and their discovery may yield new insight into this hormone-dependent cancer pathogenesis, especially endometrioid subtype.

## 11. Interaction Between the IGF1 Isoforms and Oestrogen in EC

Recent research has focused on understanding the role of specific IGF1 isoforms in cancer biology, yet their involvement in the development of EC remains inconclusive. Since endometrial tissues express both the ER and progesterone receptor (PR), the uterine lining is extremely sensitive to hormone action, which plays a major role under various physiological and pathological conditions [18,90,91]. Unopposed oestrogen exposure is a well-established risk factor for EC, whereas growth factors and related peptides are believed to mediate and modulate the autocrine/paracrine actions of hormones on their target tissues [18,92]. Imbalances in the endocrine system, such as excess oestrogen, may accelerate oncogenesis, particularly in EEC [26]. The interaction between IGF1 and oestrogen has been extensively studied in reproductive tissues, revealing complex regulatory mechanisms that govern cellular functions [7]. 

Interestingly, administration of Estradiol-17β (E2) has been shown to elevate IGF1-Ea and IGF1-Eb mRNA levels in ovariectomised mice, with a more pronounced increase observed for IGF1-Eb [93]. This upregulation is mediated by ERα through Estrogen Response Element (ERE)-dependent gene regulation, which is crucial for the full uterine response to E2 [67]. In PC3 cell lines, a human prostate cancer model commonly used to study hormonal signalling pathways, E2 modulates the expression of IGF1-Ea and IGF1-Eb while concurrently downregulating IGF1-Ec [26]. Although originating from prostate cancer, this model offers valuable insights into oestrogen-regulated IGF1 isoform expression, potentially relevant to oestrogen-sensitive tissues like the endometrium. These findings suggest that oestrogen-induced changes in IGF1 isoform expression could significantly affect uterine function and aid in developing targeted therapies for EC, particularly the EEC subtype. Stavropoulos et al. (2020) emphasised that IGF1-Ec expression may vary depending on hormonal regulation and tissue type, further supporting its potential role in oestrogen-sensitive tissues like the endometrium [86]. Additionally, another study focusing on conditions like endometriosis underscored the dynamic interplay between IGF1 isoforms and oestrogen-sensitive tissues, shedding light on hormone-regulated pathways that may also influence EC progression and therapeutic approaches [94].

## 12. Future Perspectives

Given the increasing prevalence of EC, further investigations are warranted to elucidate the endocrine interplay between oestrogen and IGF1 isoforms. A comprehensive exploration of the GH/IGF1/IGF1 isoforms axis in oestrogen-dependent EC would shed light on the distinct contributions of various IGF1 isoforms to both normal endometrial tissue development and cancer progression. These insights could inform the development of targeted IGF1R therapies, considering the well-established antagonistic effects of IGF1 isoforms on IGF1 signalling. Moreover, unravelling the molecular mechanisms underlying EC via IGF1 isoforms may pave the way for innovative diagnostic and therapeutic strategies, particularly tailored to specific subgroups of the population.

## 13. Conclusions

Although numerous studies implicate IGF1 in EC development, the evidence is complex, with conflicting findings requiring further investigation. This multifactorial and complex disease contributed to contradictory findings, including methodological inconsistencies, variances in the molecular subtypes investigated, population genetic diversity, and cancer heterogeneity. Given the expanding evidence of the significance of IGF1 isoforms in different physiology and pathophysiology, discovering these tendencies in EC seems dubious and warrants further investigation. Understanding the mechanisms governing IGF1 isoform expression in EC is essential for developing a cohesive and coherent strategy that addresses preventing, diagnosing, and treating this disease. Emerging RNA-based strategies for the treatment of EC with abnormally alternative splicing isoforms merit investigation. Additional studies are still required to address the association between alternative splicing and EC in greater depth.

## Figures and Tables

**Table 1:** IGF1 isoform expression and significant findings in a variety of cancer types.

Cancer Type	Study Type	Summary of Findings				Source
		Expression of IGF1 Isoforms			Significant Findings	
		IGF1-Ea	IGF1-Eb	IGF1-Ec		
BC	In Vitro	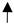	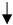	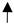	Anti-IGF1R fully inhibits IGF pro-forms, while anti-IGF1 partially inhibits their biological activity.	[75]
	In Vitro	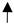	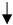	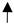 *	IGF1-Ec (hEc) enhances intracellular ERK1/2 pathway activity, promoting proliferation and migration in MCF7 cells.	[76]
	In Vitro	N/A	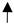 *	N/A	IGF1-Eb (hEb) acts independently of IGF-IR and enhances its biological activity against cancer cells.	[79]
CRC	In Vivo	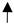	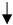	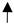	IGF1-Ea is quantitatively dominant, followed by IGF1-Eb and IGF1-Ec in CRC tissues. IGF1-Eb mRNA is significantly higher in the control large intestine compared to CRC.	[72]
	In Vivo	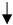	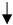	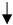	All IGF1 transcripts exhibit lower expression in CRC samples compared to controls.	[71]
	In Vitro	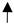	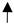	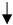	IGF1-Ea and IGF1-Eb isoforms exhibit similar high expression, while IGF1-Ec isoform expression is relatively low in DLD1 cell line.	[26]
	In Vivo	N/A	N/A	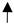	IGF1-Ec expression is higher in specimens from metastatic sites compared to primary tumours (*p* = 0.024).	[77]
CC	In Vivo	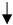	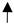	=	IGF1-Ea mRNA downregulated in both HPV-positive and HPV-negative cases compared to precancerous lesions (LSIL and HSIL with HPV-positive). IGF1-Eb is increased in CC.	[73]
	In Vitro	N/A	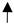 *	N/A	Synthetic IGF1-Eb (hEb) promotes HeLa cell proliferation.	[83]
	In Vitro	DOC	DOC	DOC	Viral proteins impact IGF1 gene splicing and stabilise proteins in crucial cellular processes. IGF1 isoforms exhibit differential expression across various CC cell lines.	[74]
	In Vitro	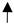	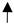	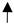	In HeLa cells, IGF1-Ea and IGF1-Eb expression levels were similar, while IGF1-Ec exhibited higher expression.	[26]
	In Vivo	N/A	N/A	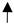	Positive cytoplasmic expression of IGF1-Ec in neuroendocrine uterine CC.	[77]
HCC	In Vitro	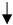	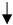	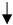	Overall IGF1 isoforms expression level lower in HepG2 cells compared to K562 cells, which might be influenced by HPV subtypes.	[74]
	In Vivo	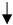	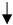	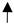	Hepatitis C virus (HCV) can alter the IGF1 splicing profile in HCC. IGF1-Ea and IGF1-Eb decreased in advance grade of HCC.	[81]
	In Vitro	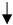	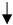	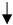	Overall IGF1 isoforms expression level lower in HuH7 cell lines.	[26]
LC	In Vitro	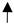 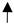	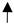	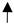	A549 cell lines exhibit elevated IGF1 isoform expression, with IGF1-Ea showing the highest levels, followed by IGF1-Ec and IGF1-Eb.	[26]
	In Vivo	N/A	N/A	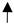	Positive cytoplasmic expression of IGF1-Ec in half of neuroendocrine LC cases.	[77]
OS	In Vitro	N/A	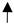 *	N/A	Synthetic hEb enhances cell growth and motility in stable U2OS cells.	[83]
	In Vitro	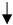	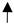	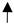	IGF1-Eb is predominantly expressed in U2OS cells compared to other cell lines (HepG2, HeLa, K562)	[74]
	In Vitro	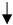	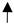	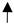	Higher expression of IGF1-Eb than IGF1-Ea in MG63 cell lines.	[26]
PC	In Vivo	N/A	N/A	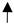 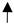	IGF1-Ec expression is higher in locally advanced tumours (stage > III).	[84]
	In Vivo and In Vitro	N/A	N/A	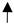	IGF1-Ec overexpressed in advanced PC; leads to EMT through ERK1/2 pathway activation and ZEB1 expression.	[78]
	In Vitro	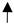	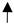	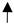 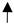	IGF-1Ea and Eb are highly expressed in lnCaP cell lines; Ec peptide reduced under hormonal conditions (oestradiol, dexamethasone and GH- treatment).	[26]
	In Vivo and In Vitro	N/A	N/A	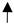 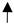 *	Synthetic human Ec peptide (hEc) stimulates the human PC3 cell growth through activating ERK1/2 pathway, without affecting Akt phosphorylation.	[85]
TC	In Vivo	N/A	N/A	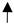 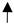	IGF1-Ec overexpressed in TC, and associated with TNM staging (prominently seen in more aggressive papillary TC) and capsule invasion of TC	[82]
GC	In Vivo	N/A	N/A	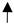 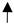	IGF1Ec peptide (MGF) was restricted to the tumour cell cytoplasm in gastric mucosa of GC cases.	[77]
PPC	In Vivo	N/A	N/A	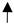 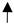	IGF1Ec peptide (MGF) was restricted to the tumour cell cytoplasm in pancreatic tissue of PPC cases.	[77]
SBC	In Vivo	N/A	N/A	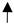 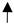	IGF1Ec peptide (MGF) was restricted to the tumour cell cytoplasm in columnar epithelium in SBC cases.	[77]
MEL	In Vitro	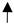 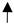	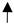	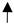	IGF1-Ea is highly expressed in human melanoma SK-MEL28 cell lines, followed by IGF1Ec and IGF1-Eb.	[26]

BC, Breast Cancer; CRC, Colorectal Cancer; CC, Epithelial Cervical Cancer; HCC, Hepatocellular Carcinoma; LC, Lung Cancer; OS, Osteosarcoma; PC, Prostate Cancer; TC, Thyroid Cancer; GC, Gastric Cancer; PCC, Pancreatic Cancer; SBC; Small Bowel Cancer; MEL, Melanoma; 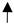, Increase expression; 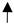

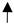
, Overexpression; 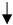, Decrease expression; P, Data reported as present; N/A, No available data; *, Action by synthetic peptide; =, Expression level is similar trend (no difference with normal/control group); DOC, Depend on cell types (cell lines).

**Table 2:** IGF1 isoforms in EC research.

Study Type	Summary of Findings	Source
In Vivo	IGF1-Ec is overexpressed in non-endometrioid carcinoma (serous papillary or clear cell carcinoma) compared to EEC and is highly expressed in areas of tumoral necrosis. It may function oppositely to PTEN, a tumour suppressor gene, while promoting tumour growth via pathways similar to survivin in EC.	[86]
In Vitro	IGF1-Ea and IGF1-Eb levels were found to be elevated in KLE cell line	[26]
In Vitro	IGF1 isoforms are expressed in KLE cells, with IGF1-Ea being most abundant. IGF1-Ec peptides stimulate KLE cell growth independently of IGF1R and INSR.	[68]

PTEN, Phosphatase and Tensin Homolog; EC, Endometrial Cancer; IGF1R, Insulin-like Growth Factor-1 Receptor; INSR, Insulin Receptor.

## Data Availability

Not applicable. This study did not generate or analyse any new data and is based solely on a review of existing literature.
